# Mitochondrial transformation occurs in cultured adipocytes, but fails to increase adipose tissue metabolic activity in mice in vivo

**DOI:** 10.1080/21623945.2022.2107178

**Published:** 2022-08-08

**Authors:** Lucia Balazova, Natalia Palesova, Miroslav Balaz

**Affiliations:** a Laboratory of Translational Nutrition Biology, Institute of Food, Nutrition and Health, ETH Zürich, Schwerzenbach, Switzerland; b Laboratory of Cellular and Molecular Metabolism, Biomedical Research Center of the Slovak Academy of Sciences, Bratislava, Slovakia

**Keywords:** Adipocyte, mitochondria, mitochondrial transformation, energy expenditure, mitochondrial respiration, metabolism

## Abstract

A large number of studies in recent years have aimed to devise novel therapeutic strategies to increase adipose tissue metabolic activity and fight the global obesity epidemics. Growing evidence suggests that cells are able to accept isolated mitochondria by a simple coincubation in a process known as mitochondrial transformation. Therefore, we aimed to test whether mitochondrial transformation occurs in mature adipocytes, and whether this phenomenon could be utilized as a therapeutic approach to increase adipose tissue mitochondrial content and improve metabolic control. We provide evidence that both brown and white adipocytes are able to rapidly accept a large amount of brown adipocyte-derived mitochondria, which remain functional for several days and significantly contribute to cellular respiration *in vitro*. However, we did not find any evidence that internalization of exogenous mitochondria would trigger transcriptional changes in the recipient cells. Moreover, injection of a large amount of brown adipocyte-derived mitochondria into the inguinal fat of C57BL/6 mice failed to increase whole-body energy expenditure, and reduce body weight gain under obesogenic conditions. This might be due to activation of immune response and rapid removal of administered mitochondria. Altogether, our study adds information on the usability of mitochondrial transformation in the treatment of metabolic disease.

## Introduction

The prevalence of obesity and associated metabolic disturbances is increasing worldwide and reaching pandemic proportions in developed countries. This excessive fat accumulation in the body is primarily caused by a long-term imbalance between energy absorption and expenditure. Numerous studies in the last decades have therefore aimed to devise novel therapeutic strategies to suppress appetite and/or increase energy expenditure. Increase in adipose tissue metabolic activity is one of the most appealing strategies to induce weight loss and improve metabolic control.

The adipose organ consists of two functionally and morphologically distinct tissue types, namely white (WAT) and brown adipose tissue (BAT) [1]. White adipocytes composed of a unilocular lipid droplet with few mitochondria are the main cell type found in WAT. In contrast, BAT is composed of adipocytes containing multiple small lipid droplets, which are rich in mitochondria and have a high oxidative capacity. Whereas WAT is mainly responsible for energy storage, metabolically active brown fat is considered the main thermogenic organ in newborn mammals and hibernating rodents [2]. This unique characteristic of BAT is enabled by the presence of uncoupling protein 1 (UCP1) on the inner mitochondrial membrane of mature brown adipocytes. UCP1 uncouples mitochondrial respiratory chain from ATP synthesis, thereby dissipating the energy of the electrochemical gradient in the form of heat [2]. Apart from the classical interscapular BAT, thermogenically active fat cells are also present within specific WAT depots [3,4]. These cells, morphologically resembling brown adipocytes, are referred to as ‘brite’ or ‘beige’ adipocytes and their formation can be induced by certain environmental, hormonal and pharmacological stimuli [3–5]. Upon cold exposure, certain WAT depots can undergo ‘browning’ most likely to support maintenance of temperature homoeostasis. This process encompasses significant changes in cellular transcriptome, morphology and functionality [5]. The most obvious morphological changes occurring during white-to-brown adipocyte conversion include fragmentation of the large lipid droplet into several smaller droplets and an increase in mitochondrial content [5,6].

Mitochondria are critical for the normal cellular function and mitochondrial dysfunction is associated with several disease states [7]. Tissues with high mitochondrial content, such as skeletal muscle, brown fat, myocardium or liver, have high energy demand. However, not only the number but also the quality of mitochondria is an important determinant of cellular metabolic activity [7,8]. Reduced mitochondrial content in WAT is associated with insulin resistance, while stimulation of mitochondrial biogenesis in white fat by PPARg agonist treatment improves whole-body insulin sensitivity and fatty acid oxidation, indicating that modulation of adipose tissue mitochondrial content and function might be a promising strategy to fight the obesity-associated metabolic disease [9,10]. Several studies have recently shown that *in vitro* cultured cells are able to accept isolated mitochondria by a simple co-incubation and there are first reports indicating therapeutic potential of this phenomenon, which was termed mitochondrial transformation [11–13]. Based on these data, we hypothesized that mitochondrial transformation might be a simple approach to increase adipose tissue mitochondrial content and metabolic activity. We could show that *in vitro* cultured adipocytes are able to accept a large number of mitochondria, which remain functional for several days and significantly contribute to cellular respiration. However, mitochondrial transformation seems to be compromised by the immune system as we could not show any increase in mitochondrial content or metabolic activity in mitochondria-injected inguinal WAT depots *in vivo*, speaking against the therapeutic potential of this approach.

## Results

### Mature brown adipocytes accept isolated mitochondria in vitro

**Figure 1.**
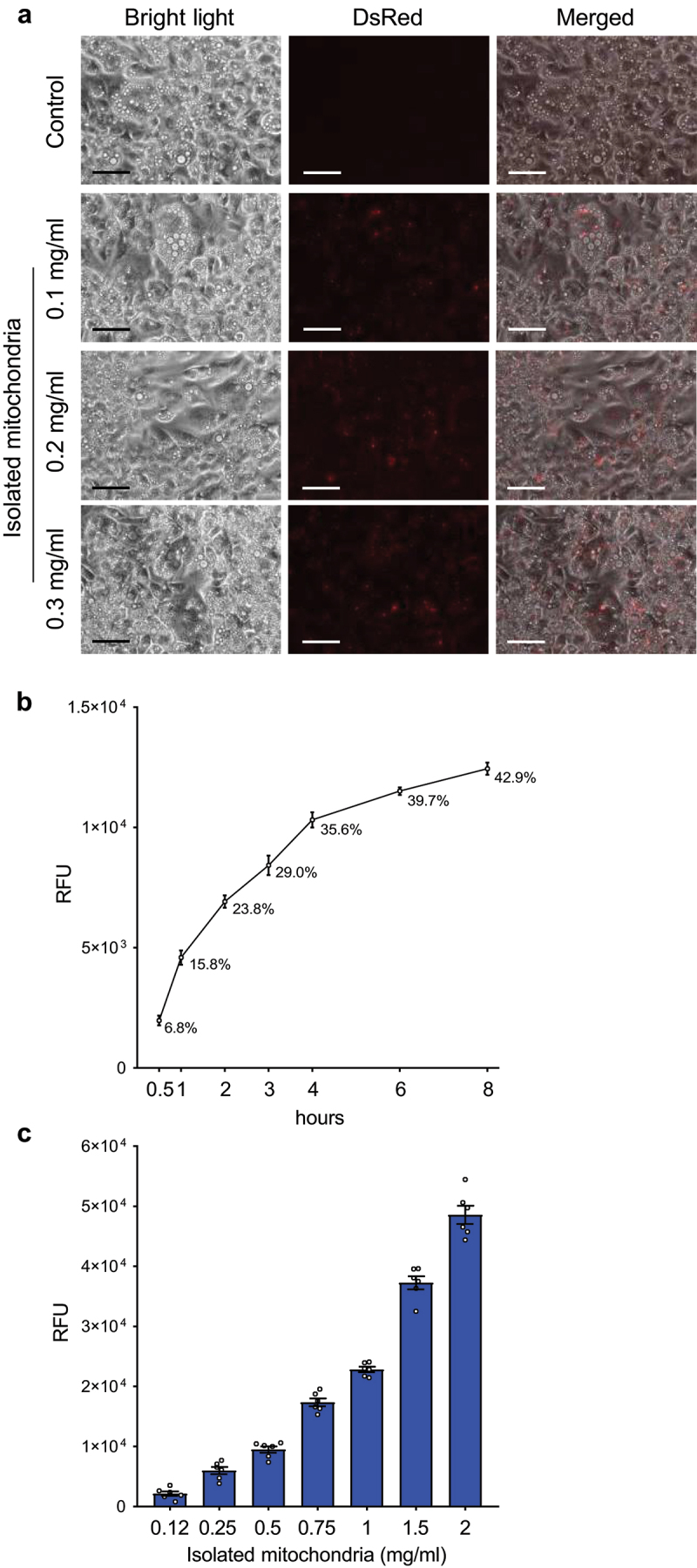
**Mature adipocytes rapidly internalize a large amount of isolated mitochondria**. (a) Mature immortalized murine brown adipocytes were incubated with DsRed2-labelled mitochondria derived from the same cell line for 24 hours. Experiment was reproduced twice. Scale bars 100 µm. (b) Time course of mitochondrial transformation into mature immortalized murine brown adipocytes. Recipient cells were incubated in the presence of murine brown adipocyte-derived DsRed2-labelled mitochondria (1 mg/ml) for indicated amounts of time. (c) Capacity of mature immortalized murine brown adipocytes to integrate mitochondria. Recipient cells were incubated in the presence of indicated amounts of DsRed2-labelled mitochondria derived from murine brown adipocytes for 24 hours, washed 5 times with PBS and fluorescence was measured after another 24 hours. Data is normalized to the protein content and expressed as mean ± SEM. N = 6 from 2 independently performed experiments.

Since multiple studies have previously shown that different cell lines are able to accept and integrate isolated mitochondria *in vitro* [11], we first aimed to test if this phenomenon occurs also in mature adipocytes. Therefore, we generated mature brown adipocytes with labelled mitochondria, using the *DsRed2-Mito7* plasmid. Transient transfection of this plasmid, which expresses a red fluorescent protein fused with the mitochondrial targeting sequence of human cytochrome C oxidase 8, allowed us to fluorescently label mitochondria in donor cells prior to their isolation. Labelled mitochondria purified from mature immortalized murine brown adipocytes were added on top of naïve mature immortalized murine brown adipocytes at different concentrations. Recipient cells were incubated in a full medium containing isolated mitochondria for 24 hours, washed five times with phosphate buffered saline (PBS) to remove non-internalized mitochondria and visualized using fluorescence microscopy. We could show a dose-dependent increase in DsRed2 signal in recipient cells, indicating that murine brown adipocytes are able to accept isolated mitochondria (Figure 1(a)). Using this approach, we could show that mitochondrial transformation also occurs in mature adipocytes.

### Mature adipocytes are able to rapidly accept a large number of mitochondria in vitro

Since we have shown that mature adipocytes can accept isolated mitochondria, we next aimed to study the dynamics of this process, as well as the capacity of adipocytes to incorporate isolated mitochondria. Therefore, we incubated naïve mature murine immortalized brown adipocytes in a full medium supplemented with DsRed2-labelled mitochondria derived from the same cell line at a concentration of 1 mg/ml. The recipient cells were washed 5 times with PBS at indicated timepoints and fluorescence was measured using a fluorescence plate reader. We could show that more than one third of mitochondria supplemented into the media were internalized by the recipient cells within the first 4 hours of co-incubation (Figure 1(b)), indicating that mitochondrial transformation is a rather rapid process. To find out the capacity of mature adipocytes to internalize mitochondria, we exposed immortalized murine brown adipocytes to increasing concentrations of DsRed2-labelled mitochondria derived from the same cell line for 24 hours, followed by extensive washing with PBS and measurement of fluorescence after another 24 hours in culture. Using this approach, we could show a dose-dependent increase in DsRed2 signal in the recipient cells (Figure 1(c)), clearly indicating that murine brown adipocytes are able to accept a large number of exogenous mitochondria. Altogether, our data indicate that *in vitro-*differentiated mature murine adipocytes are able to take up isolated mitochondria.

### Internalized mitochondria are viable and functional within recipient adipocytes in vitro

**Figure 2.**
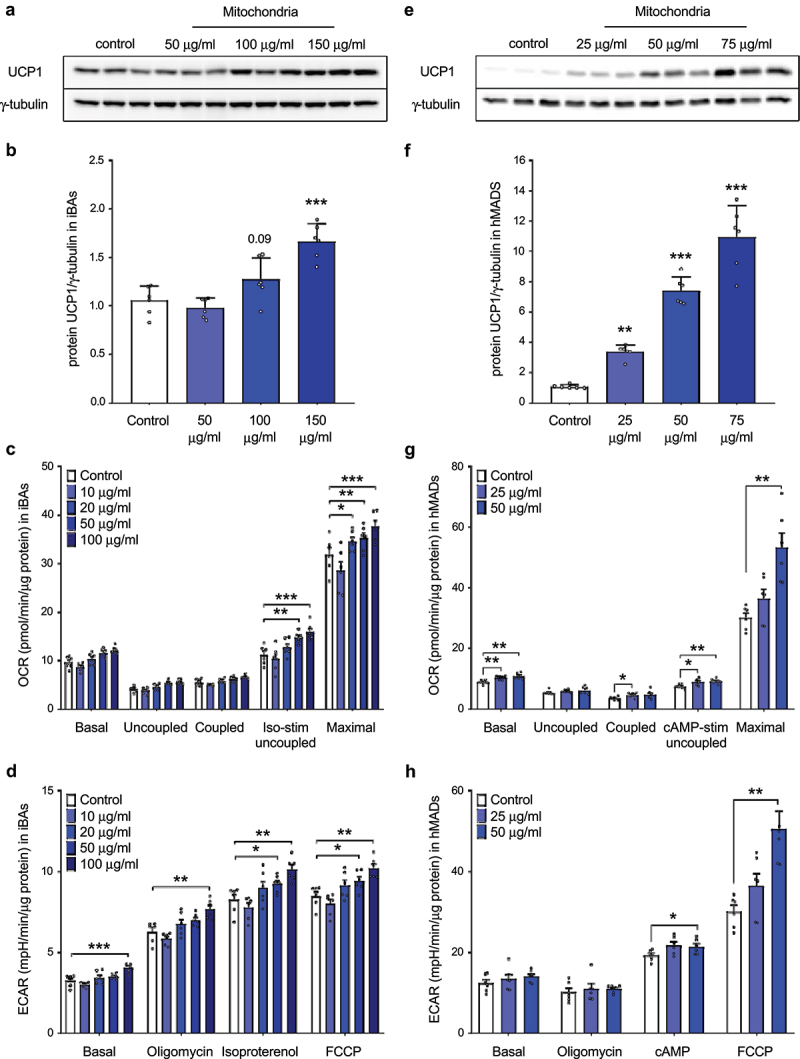
**Internalized mitochondria remain functional in recipient mature immortalized brown and human white adipocytes for several days**. (a) Representative western blot and (b) quantification of UCP1 protein, (c) mitochondrial respiration and (d) extracellular acidification rate in recipient mature immortalized murine brown adipocytes 24 hours after mitochondrial transformation. (e) Representative western blot and (f) quantification of UCP1 protein, (g) mitochondrial respiration and (h) extracellular acidification rate in recipient hMADS cells differentiated into mature white adipocytes 96 hours after transformation of human brown adipocyte-derived mitochondria. Data is expressed as mean ± SEM. N = 6 from 2 independently performed experiments. Statistical significance was calculated using ANOVA and is indicated as: *p < 0.05; **p < 0.01; ***p < 0.001.

Next, we aimed to answer the question whether the internalized mitochondria are functional and contribute to the energy metabolism of the recipient cells. Therefore, mature immortalized brown adipocytes were incubated in a full medium containing freshly isolated mitochondria derived from the same cell line for 24 hours, washed five times with PBS to remove non-internalized mitochondria and kept in culture for another 24 hours. As expected, transformation of brown adipocyte-derived mitochondria led to a dose-dependent increase in UCP1 protein level in the recipient cells (Figure 2(a) and Figure 2(b) Moreover, mitochondrial transformation caused a dose-dependent increase in isoproterenol-stimulated uncoupled and maximal mitochondrial respiration and media acidification (Figure 2(c,d)), indicating that the internalized mitochondria are functional and significantly contribute to the energy metabolism of the recipient cells. Since brown adipocytes are rich in mitochondria and show high mitochondrial respiratory rate, we hypothesized that the metabolic impact of mitochondrial transformation might be even more pronounced in white adipocytes. Therefore, we took advantage of human multipotent adipose-derived stem (hMADS) cells, which can be used to generate white and brown adipocytes from the same precursor pool. In our next experiment, hMADS cells differentiated into mature brown adipocytes serving as mitochondrial donors. Mature human white adipocytes were incubated in a full medium supplemented with isolated mitochondria for 24 hours, washed 5 times with PBS to remove non-internalized mitochondria and kept in culture for another 96 hours. To transform human hMADS cells, we used lower amount of exogenous mitochondria, as they grow at lower density and have lower mitochondrial content than immortalized murine brown adipocytes. Transformation of brown adipocyte-derived mitochondria led to a strong dose-dependent increase in UCP1 protein level in the recipient human white adipocytes (Figure 2(e, f)). Furthermore, incorporation of brown adipocyte-derived mitochondria caused a dose-dependent increase in basal, isoproterenol-stimulated uncoupled and maximal mitochondrial respiration and media acidification under stimulated conditions in the recipient human white adipocytes (Figure 2(g,h)). Altogether, these data suggest that mature human white adipocytes are able to internalize a large number of mitochondria, which remain functional for several days and significantly contribute to cellular respiration.

### Exogenous mitochondria do not trigger transcriptional changes in the recipient cells in vitro

**Figure 3.**
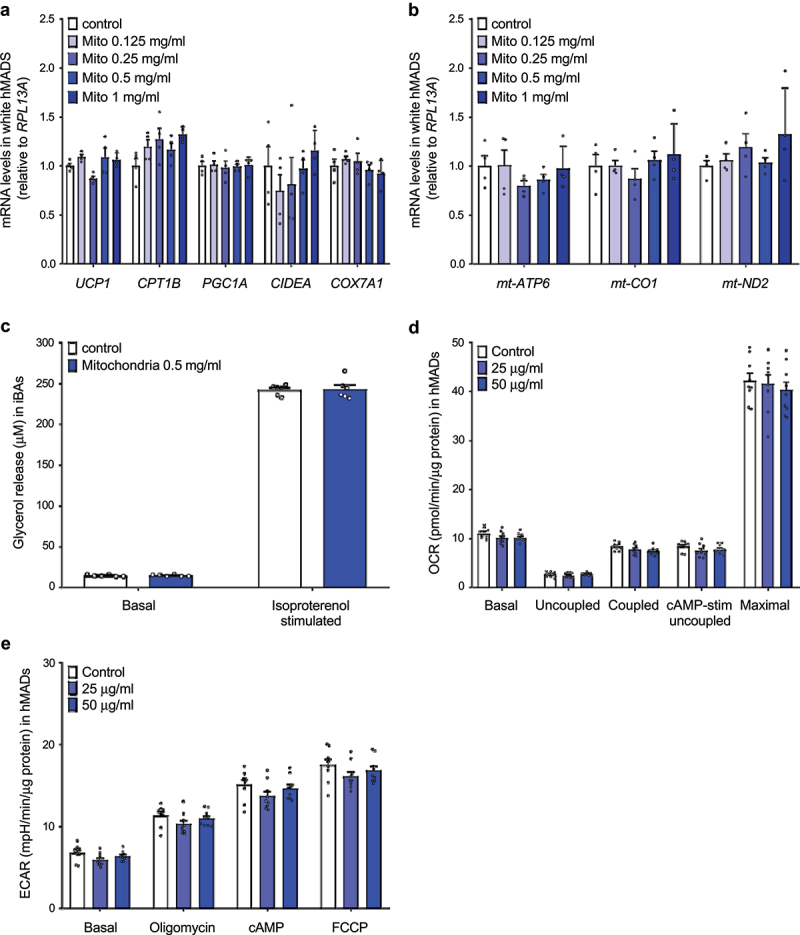
**Internalized mitochondria do not trigger transcriptional changes in recipient mature adipocytes**. (a) Expression of nuclear-encoded brown/beige adipocyte markers and (b) mitochondrially encoded genes in recipient white hMADS cells transformed with mitochondria isolated from human brown adipocytes (n = 4). (c) Glycerol release into media by immortalized murine brown adipocytes transformed with exogenous mitochondria derived from the same cell line (n = 6). (d) Oxygen consumption rate and (e) extracellular acidification rate in human white adipocytes 8 days after transformation of human brown adipocyte-derived mitochondria. Data is expressed as mean ± SEM. Experiments were reproduced twice. Statistical significance was calculated using ANOVA and T-test.

When we take into account the fact that the half-life of UCP1 protein ranges between 20 and 70 hours [14], the observed increase in UCP1 content and mitochondrial respiration 96 hours post-mitochondrial transformation indicates, that the recipient cell might be able to sense and adapt to the increased mitochondrial content. Therefore, we quantified the expression of several brown adipocyte markers, nuclear and mitochondrially encoded genes in the recipient human white adipocytes harvested 96 hours post transformation of human brown adipocyte-derived mitochondria. We did not find any effect of exogenous mitochondria on the expression of thermogenic gene markers, such as *UCP1, CPT1B, PGC1A, CIDEA* and *COX7A1* (Figure 3(a)), indicating that the increase in mitochondrial content did not promote transcriptional changes in the recipient cells. Similarly, the expression of genes encoded by mitochondrial DNA was not altered in response to mitochondrial transformation (Figure 3(b)). Moreover, we could not detect any significant effect of mitochondrial transformation on basal and isoproterenol-stimulated lipolysis in murine immortalized brown adipocytes (Figure 3(c)). Taken together, we found no evidence that the presence of exogenous mitochondria would trigger transcriptional changes in the recipient cells.

As we did not find any transcriptional changes following incorporation of exogenous mitochondria in the recipient cells, it is reasonable to assume that the observed increase in UCP1 protein content and mitochondrial respiration results from the presence of exogenous mitochondria that remain functional for several days. As the half-life of mitochondrial proteins varies from several hours to several days, we next investigated whether the observed phenotype persists for longer time periods. However, we did not find any changes in mitochondrial oxygen consumption and extracellular acidification rate in human white adipocytes 8 days after administration of exogenous mitochondria originating from the same cell line (Figure 3(d,e)). These data suggest, that the positive effect of mitochondrial transformation on metabolic activity of adipocytes is only short term.

### Mitochondrial transformation does not increase energy expenditure in C57BL/6 mice

**Figure 4.**
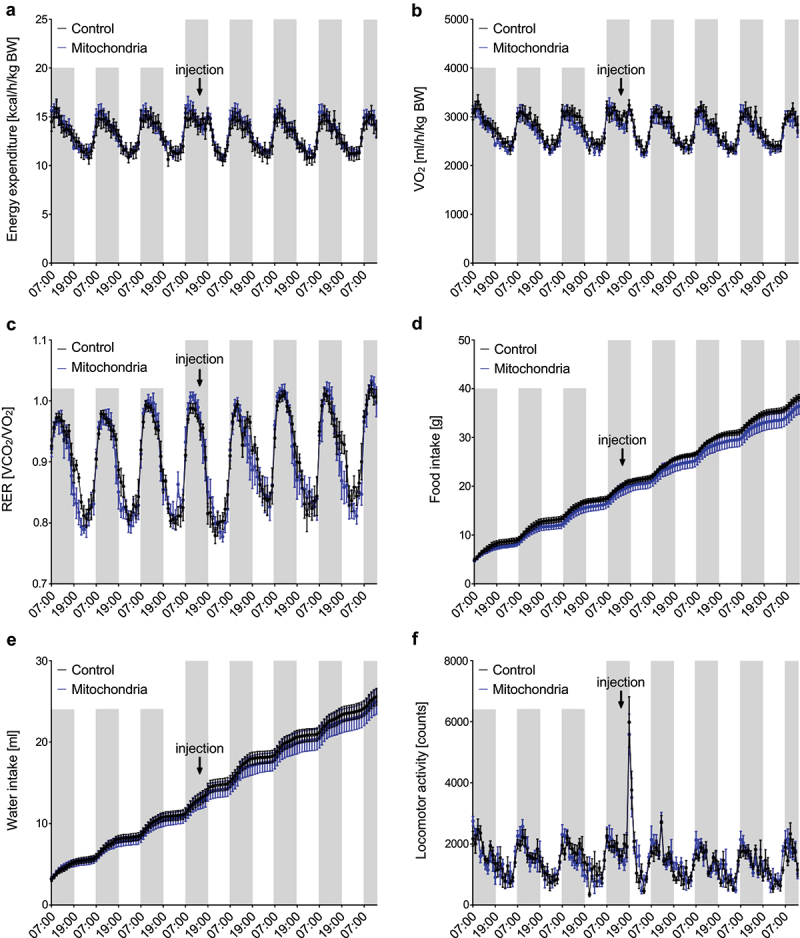
**Administration of exogenous mitochondria into inguinal WAT does not increase energy expenditure in mice *in vivo.*** Administration of immortalized murine brown adipocyte-derived mitochondria does not affect (a) whole-body energy expenditure, (b) oxygen consumption, (c) respiratory exchange ratio (RER), (d) food and (e) water intake or (f) locomotor activity in C57BL/6 mice (n = 5). Data is expressed as mean ± SEM. Statistical significance was calculated using a T-test.

To test the therapeutic potential of mitochondrial transformation, we used *in vitro* differentiated murine brown adipocytes as donors of mitochondria. Mitochondria isolated by differential centrifugation from two P10 dishes (approximately 20 million cells) were resuspended in a respiration buffer and injected into both inguinal white adipose tissue depots (10 mg per depot) of 12-week-old male C57BL/6 mice, while control littermates were injected with the same volume of respiration buffer. Administration of purified mitochondria did not affect whole-body energy expenditure, oxygen consumption, respiratory exchange ratio, food and water intake, or locomotor activity in wild-type C57BL/6 mice (Figure 4(a-f)).

***Mitochondrial transformation neither affects UCP1 protein and mitochondrial content in iWAT, nor body weight gain in C57BL/6 mice***

**Figure 5.**
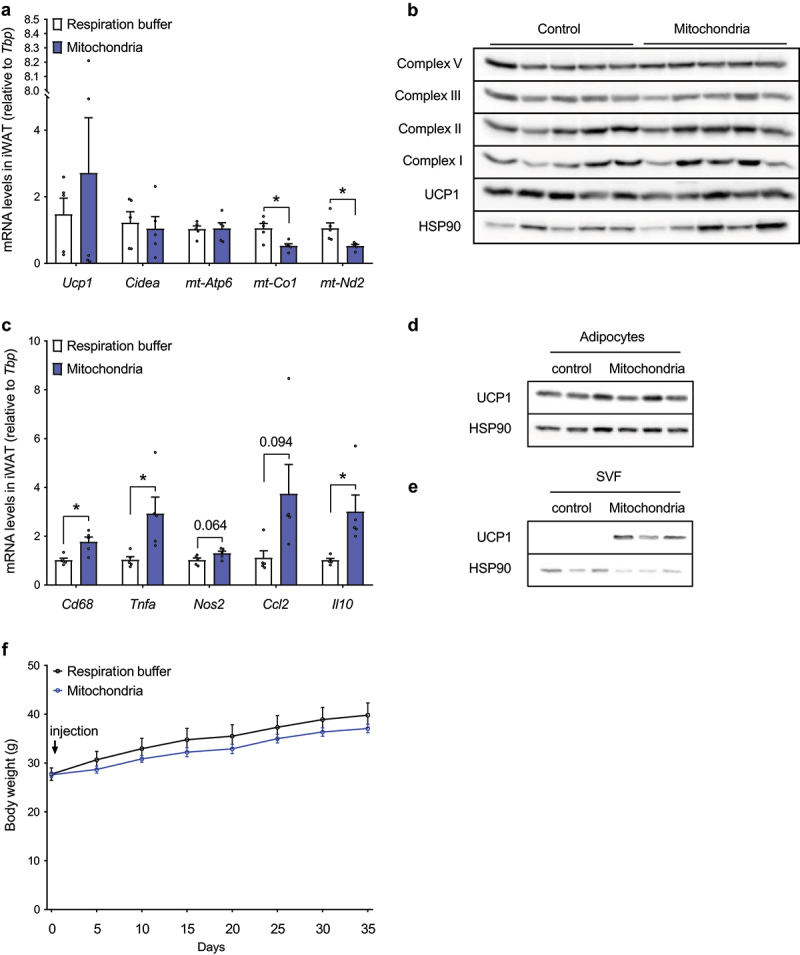
**Administration of exogenous mitochondria neither affects UCP1 and mitochondrial content in inguinal WAT, nor body weight gain under obesogenic conditions *in vivo.*** (a) Expression of thermogenic and mitochondrially encoded genes in iWAT of C57BL/6 mice 6 days after injection of respiration buffer or freshly isolated brown adipocyte-derived mitochondria (20 mg; 10 mg per depot; n = 5). (b) Immunostaining of UCP1 and individual OXPHOS complexes in iWAT of C57BL/6 mice 6 days after injection of respiration buffer or isolated brown adipocyte-derived mitochondria (20 mg; 10 mg per depot; n = 5). (c) Expression of inflammatory gene markers in iWAT of mice 6 days after injection of respiration buffer or freshly isolated brown adipocyte-derived mitochondria (20 mg; 10 mg per depot; n = 5). UCP1 protein content in adipocyte-enriched fraction and (e) stromal vascular fraction of inguinal white adipose tissue 48 hours after administration of immortalized murine brown adipocyte-derived mitochondria (1 mg per depot). (f) Body weight gain in mice injected with respiration buffer or isolated brown adipocyte-derived mitochondria (15 mg per iWAT depot) on high fat diet (n = 5). Data is expressed as mean ± SEM. Statistical significance was calculated using a T-test and is indicated as: *p < 0.05.

Since we could not observe any effect of mitochondrial injection on energy expenditure, we focused on molecular analysis of iWAT depots. Immortalized brown adipocyte-derived mitochondria were injected into iWAT depots of 14-week-old male C57BL/6 mice (10 mg per depot), while control littermates were injected with the same volume of respiration buffer. Tissues were harvested 6 days post injection and used for quantification of brown adipocyte markers and mitochondrially encoded genes and proteins. Injection of isolated mitochondria did not affect *Ucp1, Cidea* and *mt-Atp6* mRNA levels, while *mt-Co1* and *mt-Nd2* expressions in iWAT were significantly reduced (Figure 5(a)). Similarly, the amount of UCP1 protein and individual complexes of mitochondrial respiratory chain in iWAT was not affected (Figure 5(b)), clearly indicating that the injected brown adipocyte-derived mitochondria were not internalized and integrated by mature adipocytes. Therefore, we also quantified the expression of inflammatory gene markers in iWAT depots, and we found a significant increase in *Cd68, Tnfa* and *Il10*, while *Nos2* and *Ccl2* showed a trend towards an increase (Figure 5(c)). These data indicate that administration of exogenous mitochondria triggers an inflammatory response in iWAT, and injected mitochondria are most likely removed by activated macrophages. To further address this point, we performed inguinal adipose tissue fractionation 48 hours after injection of mitochondria (1 mg per depot) isolated from immortalized murine brown adipocytes. In agreement with our previous observations, significant amounts of UCP1 protein could be detected in the stromal vascular fraction, further supporting clearance of exogenous mitochondria by the immune cells (Figure 5(d,e)).

We also tested the effect of exogenous mitochondria administration on body weight gain in mice challenged with a high fat diet. Both iWAT depots of 12-weeks old C57BL/6 mice were injected either with freshly isolated brown adipocyte-derived mitochondria (15 mg per depot), or with the same volume of respiration buffer. Mice were exposed for 5 weeks to a high fat diet feeding regime and body weight gain was recorded every 5 days. In agreement with the energy expenditure and tissue analysis data, we could not detect any significant effect of exogenous mitochondria administration on body weight gain under obesogenic conditions (figure 5(f)). Altogether, these data suggest that even though the phenomenon of mitochondrial transformation occurs in cultured mature adipocytes *in vitro*, administration of isolated mitochondria fails to increase WAT metabolic activity in mice *in vivo*.

## Discussion

Since white adipose tissue is one of the largest organs in human body accounting for more than 20% of body mass in obese individuals, even a modest increase in adipocyte metabolic activity might have a profound effect on whole-body energy expenditure and metabolic control. This can be achieved either by an increase in mitochondrial content, functionality or both. Here, we show that the phenomenon of mitochondrial transformation also occurs in *in vitro* differentiated mature brown and white adipocytes. In fact, we could show that adipocytes are able to rapidly internalize a large number of isolated mitochondria, which remain functional for several days and significantly contribute to cellular respiration.

Already in 1982 it was shown that isolated mitochondria can be incorporated *in vitro* into mammalian cells by a process termed mitochondrial transformation [15]. Recently, it was shown that internalization of mitochondria into recipient cells is a rapid process, which seems to be mediated by micropinocytosis and requires intact outer mitochondrial membrane, which we could confirm also in our study on mature adipocytes. Internalized mitochondria are viable, co-localize with endogenous mitochondria and contribute to cellular metabolic activity [11]. Injection of isolated mitochondria into the myocardium of rabbits and pigs could protect the heart from ischaemia-reperfusion injury by enhancing oxygen consumption, high-energy phosphate synthesis and induction of pathways that are important for preserving myocardial energetics and cell viability [12,13]. Moreover, in a pilot clinical study mitochondrial transformation was employed in paediatric patients suffering from myocardial ischaemia. All five patients showed qualitative improvement in left ventricular function within days, without short-term complications [16]. However, these results have to be interpreted with caution due to the absence of a proper control group and a very low number of participants.

The concept of mitochondrial transformation as a therapeutic approach to treat mitochondrial dysfunction-associated disorders is quite controversial [17,18]. On the one hand, administration of mitochondria isolated from *in vitro* cultured cells would be a very simple and elegant way to increase mitochondrial content and restore mitochondrial function. On the other hand, there are several critical points that need to be taken into account when considering the therapeutic use of this approach. (1) Even though there is no doubt that *in vitro* cultured cells are able to accept isolated mitochondria, their functionality remains questionable. Integrated mitochondria are not associated with endosomal or autophagic structures, but are co-localized with endogenous mitochondria [11]. Functionality of integrated mitochondria was demonstrated by the restoration of mitochondrial respiration in human Rho0 cells, which lack functional mitochondria [19]. Other studies have shown increased oxygen consumption and ATP levels in the recipient cells [12,20]. We could confirm that transformation of brown adipocyte-derived mitochondria leads to a dose-dependent increase in UCP1 protein levels and mitochondrial respiration in mature white and brown adipocytes. We assume that a higher content of functional mitochondria would trigger an adaptive response in the recipient cells, in terms of substrate availability and utilization, and expression of mitochondrial proteins encoded by genomic and mitochondrial DNA. Therefore, it is surprising that we could not detect any increase in basal and isoproterenol-stimulated lipolysis, expression of *UCP1* mRNA and mitochondrially encoded genes in the recipient white adipocytes 96 hours post transformation of brown adipocyte-derived mitochondria. This is very interesting especially when we take into account the fact that the half-life of UCP1 protein ranges between 20 and 70 hours in stimulated and non-stimulated brown adipocytes, respectively [14]. In addition, we observed only a modest increase in uncoupled mitochondrial respiration in white adipocytes despite a massive increase in UCP1 content. More studies are needed to address the functionality of internalized mitochondria in the recipient cells.

(2) Another critical point is the relatively short half-life of mitochondria, which is tissue-specific and varies between 2 days in the liver and 14 days in the heart muscle [21,22]. Since mitochondrial transformation does not trigger any major transcriptional changes to increased mitochondrial content in the recipient cells, it is reasonable to assume that even if the exogenous mitochondria were taken up by the recipient cells and increased the metabolic activity of the target tissue, the effect would be only transient. This is supported by our in vitro data showing that mitochondrial respiration is increased in human white adipocytes 4 days after mitochondrial transformation, but this effect disappears at later timepoints. Furthermore, in our study, we could not detect any significant effect of mitochondrial injection into inguinal WAT on whole-body energy expenditure, substrate preference and body weight gain under obesogenic conditions in C57BL/6 mice, even though we looked at very early timepoints following administration of large amounts of mitochondria. One explanation might be the impairment of mitochondrial viability in the extracellular fluid. This is also supported by the fact that UCP1 and OXPHOS protein levels in iWAT were not altered by administration of exogenous brown adipocyte-derived mitochondria.

(3) In all published studies, freshly isolated mitochondria were resuspended in standard Ca^2+^-free respiration buffer, to prevent Ca^2+^-induced formation of mitochondrial transition pores in the inner membrane and leakage of mitochondrial content [23,24]. Subsequently, the mitochondrial suspension was injected into myocardium, coronary arteries [16,25,26] or inguinal WAT in our study, exposing mitochondria to high Ca^2+^ concentrations in the blood or the extracellular fluid. Therefore, it is questionable whether isolated mitochondria could remain viable after injection into the tissue or blood stream [18]. However, high calcium concentration is not the only major difference between cell culture media and extracellular fluid. (4) Even though we and others have shown that mitochondrial transformation is a rather rapid process and cells are able to accept a large amount of mitochondria [11], it takes several hours until the majority of administered mitochondria get internalized by the recipient cells. When injected into the tissue, integration of isolated mitochondria might be compromised by the immune cell defence. Indeed, we have shown that administration of exogenous mitochondria triggers an inflammatory response in inguinal WAT, as expression of proinflammatory macrophage markers was significantly increased. Moreover, already 6 days post injection we could not detect any increase in UCP1 protein and individual complexes of mitochondrial respiratory chain, despite administration of a large amount of freshly isolated brown adipocyte-derived mitochondria. Therefore, we believe that the viability of administered mitochondria might have been compromised by the presence of high calcium concentrations and/or activated immune cells in the extracellular environment. Our hypothesis is supported by the finding of significant amounts of UCP1 protein in the stromal vascular fraction of mitochondria-injected iWAT.

Altogether, even though we could confirm that mitochondrial transformation occurs under *in vitro* settings also in mature adipocytes, our *in vivo* results along with the above-mentioned arguments clearly speak against the therapeutic potential of mitochondrial transformation in increasing adipose tissue metabolic activity and improving metabolic control. Further studies are needed to address the viability and functionality of exogenous mitochondria in the recipient cells.

## Study limitations

Our study consistently shows that the process of mitochondrial transformation occurs in cultured brown and white adipocytes *in vitro* but fails to increase metabolic activity of adipocytes in mice *in vivo*. However, there are several limitations that need to be considered. Even though we used a standardized protocol for isolation of intact and functional mitochondria, our study lacks functional characterization and quantification of purity of isolated mitochondria. The functionality of isolated mitochondria is supported by the finding that administration of exogenous mitochondria dose-dependently promotes mitochondrial respiration in the recipient cells. The amount of mitochondria injected into inguinal white adipose tissue was selected based on a pilot experiment, in which all tested amounts (1, 5, 10 and 30 mg; data not shown) failed to achieve an increase in UCP1 protein content. Therefore, we decided to perform the experiments with higher concentrations of administered mitochondria to increase the chance that some of the exogenous mitochondria will escape the immune system and will get internalized by mature adipocytes. In the present study, we used a mouse model with diet-induced obesity to examine the effect of mitochondrial transformation on the metabolic phenotype. Future studies on genetic mouse models of obesity are needed to test the therapeutic potential of this approach.

## Material and methods

### Mouse experiments

All animal procedures were approved by the Veterinary office of the Canton of Zürich. All the mice used for the experiments were male, housed 3–4 littermates per cage in individually ventilated cages in standard housing conditions (22°C, 12 h reversed light/dark cycle, dark phase starting at 7 am), with ad libitum access to chow (18% proteins, 4.5% fibres, 4.5% fat, 6.3% ashes, Provimi Kliba SA) and water. 12–14 weeks old male C57BL/6 mice (Charles River) were subjected to subcutaneous injection of mitochondria into inguinal WAT depot. In high fat diet cohort (23.9% proteins, 4.9% fibres, 35% fat, 5.0% ashes, Provimi Kliba SA), the feeding regimen (5 weeks) was initiated at the age of 12 weeks.

### Indirect calorimetry

Indirect calorimetry measurements were performed with the PhenoMaster (TSE Systems) according to the manufacturer’s guidelines. O_2_ and CO_2_ levels were measured for 60 s every 13 minutes continuously. Energy expenditure was calculated according to the manufacturer’s guidelines. The respiratory quotient was estimated by calculating the ratio of CO_2_ production to O_2_ consumption. The animals were single-caged and acclimated to the metabolic cage for 48 hours prior to metabolic recording. Locomotor activity, food and water intake were monitored throughout the whole measurement.

### Tissue harvest

Animals were euthanized singly in carbon dioxide atmosphere. All tissues were carefully dissected, weighed and snap frozen in liquid nitrogen until further processing. Popliteal lymph nodes were carefully removed from iWAT for all gene and protein expression analyses. For RNA and protein isolation, whole adipose tissue depot was homogenized. For isolation of floating mature adipocytes and stromal-vascular fraction (SVF), the inguinal white adipose tissue depots were cut into smaller pieces, collagenase digested and individual fractions were separated by differential centrifugation. Both the supernatant containing adipocyte enriched fraction and the pellet containing SVF cells were washed three times to remove cell debris and any non-internalized mitochondria, and lysed in RIPA buffer for protein analysis by immunoblot.

### Cell culture – hMADS cells

HMADS cells originating from the prepubic fat pad of a 4-month-old male were kindly provided by Dr. Amri and cultured as previously described [27]. Briefly, cells (between passage 14 and 16) were grown in low glucose DMEM supplemented with 15 mM HEPES, 10% FBS, 2 mM L-glutamine, 1% Penicillin/Streptomycin and 2.5 ng/ml recombinant human FGF-2 (Peprotech) in normoxic humidified cell culture incubator (5% CO2 and 37°C). The medium was changed every other day, and FGF-2 was omitted after cells reached confluence. Differentiation of 48 hours post-confluent cells was induced (day 0) by a maintenance medium (DMEM/Ham’s F12 media (Lonza) containing 10 µg/ml Transferrin, 10 nM insulin and 0.2 nM triiodothyronine) supplemented with 1 µM dexamethasone and 500 µM isobutyl methylxanthine (IBMX) and from day 2 to 9, cells were cultured in maintenance medium containing 100 nM rosiglitazone. Cells were kept in culture until day 18 in absence of rosiglitazone to obtain mature white adipocytes. To obtain brown adipocytes, cells were exposed to an additional rosiglitazone pulse between days 14–18. Mitochondrial transformation was performed on day 13 in the maintenance medium and after 24 hours, cells were washed 5 times with PBS and kept in culture for another 96 hours. Adipocytes were cultured until day 18, when cellular respiration was determined, or cells were harvested for RNA and protein analysis. All cell lines used were regularly tested negative for mycoplasma contamination throughout the whole duration of this study.

### Cell culture – immortalized murine brown adipocytes

Preadipocytes isolated from the iBAT stromal-vascular fraction of late foetal and newborn C57Bl/6 mice (both genders) and immortalized by introducing the SV40 antigen were kindly provided by Prof. Klein [28]. Preadipocytes (between passage 4 and 6) were grown on collagen-coated plates in DMEM containing 10% FBS and 1% Pen/Strep (Gibco) in normoxic humidified cell culture incubator (5% CO2 and 37°C). After reaching confluence, adipogenic differentiation was induced by supplementing the medium with IBMX (500 μM), dexamethasone (1 µM), insulin (20 nM), T3 (1 nM) and indomethacin (125 μM). All compounds were obtained from Sigma-Aldrich (specification in Key Resources Table). After 48 hours, medium was replaced by a fresh maintenance medium containing (insulin and T3), which was replaced every other day. For mitochondrial transformation, differentiating adipocytes (day 5) were trypsinized, counted and replated on collagen-coated multi-well plates to reduce cell density. Cells were allowed to attach, recover and maturate before mitochondria were supplemented in the media (day 7). Cells were harvested on day 8–9 for RNA and protein analysis, or cellular respiration measurements. All cell lines used were regularly tested negative for mycoplasma contamination throughout the whole duration of this study.

### Mitochondrial isolation and transformation

To obtain mitochondria, cultured cells were fractionated using differential centrifugation. Mature immortalized murine brown adipocytes (day 8–9) and human hMADS cells-derived brown adipocytes (day 17) cultured on P10 dishes were washed 3 times with ice-cold PBS and scraped in fractionation buffer (250 mM Sucrose, 20 mM HEPES (pH 7.4), 10 mM KCl, 1.5 mM MgCl2, 1 mM EDTA, 1 mM EGTA) supplemented with 1 mM DTT (Sigma-Aldrich). Cells were passed through a 25-gauge needle 10 times. In the first centrifugation step (500 g/5 min/4°C) nuclei were pelleted. Supernatant containing cytosol, membranes and organelles was transferred into a clean Eppendorf tube and centrifuged (3000 g/5 min/4°C) to pellet mitochondria. After washing 3 times with fresh fractionation buffer, the wet mitochondrial pellet was weighed and resuspended in DMEM for *in vitro* use, or in fresh respiration buffer (250 mmol/l sucrose, 2 mmol/l KH_2_PO_4_, 10 mmol/l MgCl_2_, 20 mmol/l HEPES buffer, pH 7.2, 0.5 mmol/l EGTA, pH 8.0, 5 mmol/l glutamate, 5 mmol/l malate, 8 mmol/l succinate, and 1 mmol/l ADP) for *in vivo* application. For fluorescent labelling of mitochondria, DsRed2-Mito7 plasmid (Addgene plasmid # 55838) was transfected into mature adipocytes on day 5 using TurboFect (Dharmacon). From a single P10 dish of mature immortalized murine brown adipocytes (~10–12 million cells/dish) and human hMADS cells-derived brown adipocytes (~4–5 million cells/dish) we were able to obtain approximately 10 and 5 mg (wet weight) of isolated mitochondria, respectively.

## Mitochondrial uptake

Mitochondrial uptake (fluorescence) in recipient cells was measured using a plate reader. After indicating incubation times in the presence of purified mitochondria, cells were thoroughly rinsed (5 times) with ice-cold PBS to remove all non-internalized mitochondria and fluorescence of native (no fixation) cells was measured. After measuring fluorescence, recipient cells were lysed in RIPA buffer. Protein content was estimated and used for data normalization. The data is expressed as the percentage of mitochondria taken up (relative to the total administered mitochondria) at indicated timepoints. Measurement of fluorescence in wells containing recipient cells, but no exogenous mitochondria, was used as blank.

### Cellular respiration

To measure cellular respiration, immortalized murine brown adipocytes were cultured on collagen-coated cell culture dishes. Differentiating adipocytes were trypsinized and replated on day 5 at density 7.000 cells per well and allowed to recover for 48 hours before treatment. Since hMADS cells grow in a monolayer, they are differentiated directly on collagen-coated 96-well Seahorse microplates. On the day of the experiment, the adipogenic medium was replaced with XF Assay Medium (pH 7.4, Seahorse Bioscience) supplemented with glucose (1 g/L; Sigma-Aldrich), 2 mM sodium pyruvate (Invitrogen) and 2 mM L-glutamine (Invitrogen). The oxygen consumption rate (OCR) was measured using the Extracellular flux analyser XF96 (Agilent). Test compounds were sequentially injected to obtain the following concentrations: 1 µg/ml Oligomycin, 1 µM isoproterenol (0.5 mM dibutyryl cAMP for hMADS), 1 µg/ml FCCP, 3 µM Rotenone with 2 µg/ml Antimycin A. OCR and ECAR levels were normalized to protein amount per well (µg protein). Non-mitochondrial respiration was subtracted to obtain basal, basal uncoupled, stimulated uncoupled and maximal mitochondrial respiration.

### Lipolysis

Lipolytic activity of mature brown adipocytes was determined as glycerol release into culture media. Briefly, the cells were serum starved for 2 hours in a low-glucose medium (Gibco) prior to the analysis. Isoproterenol (1 µM; Sigma-Aldrich) was added, and the plate was incubated for another 30 minutes at 37°C in a humidified CO_2_ incubator. Media were collected and spun down into pellet detached cells. Glycerol was assessed in the supernatant using the Glycerol reagent (Sigma-Aldrich) according to manufacturer’s instructions.

### RNA extraction, cDNA synthesis, quantitative RT-PCR

Total RNA was extracted from tissues or cells using Trizol reagent (Invitrogen) according to the manufacturer’s instructions. DNase treatment (NEB Biolabs) was included to remove traces of genomic DNA. Reverse transcription was performed to generate cDNA library by using the High-Capacity cDNA Reverse transcription kit (Applied Biosystems) with 1 μg of RNA. Quantitative PCR was performed on a ViiA7 (Applied Biosystems) and relative mRNA concentrations normalized to the expressions of *RPL13A1* and *TBP* were calculated by the ∆∆Ct method. All primers used in this study are listed in the Supplemental Table.

### Protein extraction and Western blot

Adipose tissue samples and *in vitro* differentiated adipocytes were homogenized in RIPA buffer (50 mM Tris-HCl pH 7.4, 150 mM NaCl, 2 mM EDTA, 1.0% Triton X100, 0.5% sodium deoxycholate) supplemented with protease (Complete, Roche) and phosphatase (Halt phosphatase inhibitor cocktail, ThermoFisher) inhibitor cocktails. Lysates were cleared by centrifugation at 12.000 g for 15 minutes at 4°C. Protein concentration of the supernatants was determined by DC Protein Assay (Bio-Rad). Equal protein amounts (5–20 µg) were separated on a 12% SDS-polyacrylamide gel, transferred to a nitrocellulose membrane (Bio-Rad) and stained for UCP1 (1:1000, Pierce), OXPHOS (1:1000, Abcam), HSP90 (1:1000, Cell Signalling) and γ-tubulin (1:10.000, Sigma-Aldrich). Signal of the HRP-conjugated secondary antibodies (1:10.000, Calbiochem) was visualized by the ImageQuant system (GE Healthcare Life Sciences).

## Data Availability

All data presented in the manuscript are available under: https://data.mendeley.com/datasets/pwgc8z9zdm/2
